# The Role of Forkhead Box Family in Bone Metabolism and Diseases

**DOI:** 10.3389/fphar.2021.772237

**Published:** 2022-01-28

**Authors:** Jianxiang Xu, Kanbin Wang, Zengjie Zhang, Deting Xue, Weixu Li, Zhijun Pan

**Affiliations:** ^1^ Department of Orthopedic Surgery, The Second Affiliated Hospital, Zhejiang University School of Medicine, Hangzhou, China; ^2^ Orthopedics Research Institute of Zhejiang University, Hangzhou, China; ^3^ Key Laboratory of Motor System Disease Research and Precision Therapy of Zhejiang Province, Hangzhou, China; ^4^ Department of Orthopedic Surgery, The Fourth Affiliated Hospital, Zhejiang University School of Medicine, Yiwu, China

**Keywords:** Fox family, bone metabolism, bone diseases, signaling pathways, Fox factors-based therapeutics

## Abstract

Forkhead box (Fox) family, an evolutionarily conserved family of transcription factors carrying the “Forkhead” motif, plays an indispensable role in human health and disease. Fox family genes are involved in cell differentiation, proliferation and apoptosis, embryonic development, aging, glucose and lipid metabolism, and immune regulation. The regulatory role of the Fox family in the context of bone metabolism and orthopedic diseases is an emerging research hotspot. In this review, we highlight the major molecular mechanisms underlying the regulatory role of Fox factors in bone metabolism, bone development, bone homeostasis, and bone diseases associated with inhibition or upregulation of Fox factors. In addition, we discuss the emerging evidence in the realm of Fox factor-based therapeutics.

## 1 Background

Fox family, identified in 2000 (Kaestner et al., 2000), is a group of genes with “Forkhead” motif-dependent transcription factors. Recent studies have unraveled the role of Fox family genes as key sensors for bone metabolism. Members of the Fox family respond to metabolic stress in bone tissue, inflammatory signals, hypoxic/oxidative stress, and are associated with aging and autophagy.

The role of Fox in the regulation of bone metabolism was first recognized in 2001 when a defective reproductive phenotype was identified as a molecular marker of prospective rib cartilage (Sudo et al., 2001). Since then, the role of different subfamilies of Fox in bone metabolism, from FoxA to FoxS (based on the degree of homology in their forkhead domains), has been investigated. Over the years, our knowledge of the role of Fox in bone metabolism has grown exponentially along with the awareness of the key roles of Fox-regulated biological processes in bone functions (Huang et al., 2020).

Studies have demonstrated differential expression of Fox factors in osteoblasts in the setting of skeletal disease compared with normal osteoblasts; these differentially expressed factors have been shown to promote or suppress the development of osteoporosis by regulating bone metabolism (Greenblatt et al., 2010; Niedan et al., 2014; Yu et al., 2014; Guan et al., 2015; Hopkins et al., 2016; Zeng et al., 2017). Bone metabolism refers to a complex series of biological processes involving multiple signaling pathways, such as Wingless and Int-1 (Wnt)/β-catenin pathway (Huang et al., 2020), bone morphogenetic protein (BMP)/drosophila mothers against decapentaplegic (Smad) pathway (Guan et al., 2015), phosphatidylinositol 4,5-bisphosphate 3-kinase (PI3K)/Akt pathway (Hopkins et al., 2016), transforming growth factor-β (TGF-β) pathway (Niedan et al., 2014), p38/mitogen-activated protein kinase (MAPK) pathway (Zeng et al., 2017), and nuclear factor-kappa B (NF-κB) pathway (Greenblatt et al., 2010). Fox factors are involved in the regulation of bone metabolism, either directly or by acting as downstream effectors of these signaling pathways (Greenblatt et al., 2010; Niedan et al., 2014; Yu et al., 2014; Guan et al., 2015; Hopkins et al., 2016; Zeng et al., 2017). In addition to its role in osteoporosis, Fox factors are also involved in the development of osteoarthritis (Charlier et al., 2016), rheumatoid arthritis (Reedquist et al., 2006; Wasén et al., 2020), intervertebral disc degeneration (Alvarez-Garcia et al., 2018), and bone tumors (Nakamura et al., 2000; Cidre-Aranaz and Alonso, 2015; Haider et al., 2016). In particular, drugs targeting Fox factors have been reported to inhibit the progression of bone tumors (Lam and Gomes, 2014). Last but not the least, mutations in Fox factor have been implicated in inherited skeletal abnormalities (Seifi and Walter, 2018; Chen et al., 2019).

**FIGURE 1:**
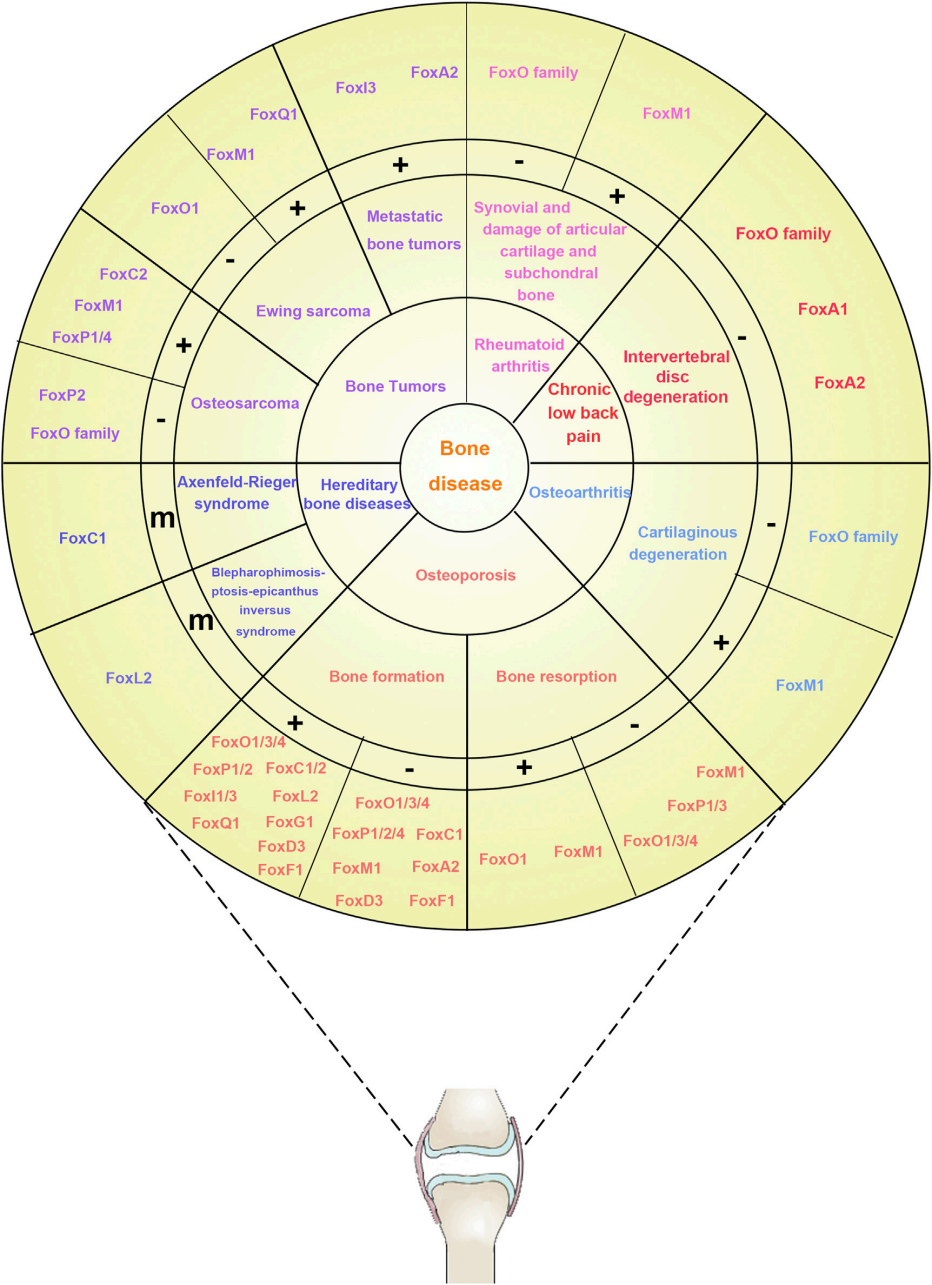
The relationship between the Fox family and bone diseases. The bone diseases related to the Fox family are divided into six types listed in the first inner ring (six types of colors, including osteoporosis, osteoarthritis, chronic low back pain, rheumatoid arthritis, bone tumors, and hereditary bone diseases). The characteristics of different diseases are listed in the second inner ring. Osteoporosis, a metabolic bone disease caused by dyshomeostasis of bone metabolism, is closely related with bone formation and bone resorption; Osteoarthritis, a chronic joint disease characterized by degenerative changes in joint cartilage, is closely related with cartilaginous degeneration; Chronic low back pain is closely related with intervertebral disc degeneration; Rheumatoid arthritis, a chronic joint disease characterized by persistent synovitis and associated damage to the articular cartilage and subchondral bone, is closely related with synovial and damage of articular cartilage and subchondral bone; Bone tumors is separated into metastatic bone tumors (a subtumor formed in bone originated from other parts of body), Ewing sarcoma (a rare and highly aggressive cancer that occurs primarily in the bones and surrounding tissues of children and adolescents), and osteosarcoma (the most common primary malignant tumor of bone, and it occurs mainly in children and adolescents); Hereditary bone diseases, caused by genetic factors, is separated into Axenfeld–Rieger syndrome and blepharophimosis-ptosis-epicanthus inversus syndrome. The correlations between the characteristics of different diseases and Fox-related genes are listed in the third inner ring. “+,” Fox-related genes enhance this symptom (or activity); “−,” Fox-related genes inhibit this symptom (or activity); “m,” mutation of Fox-related genes. The Fox-related genes are listed in the outer ring.

Although the close relationship between Fox factor and skeletal disease is well established, the role of Fox factors in promoting or inhibiting skeletal disease and the associated underlying mechanisms are highly controversial and perplexing. In this review, we summarize the available evidence of the functional role of the Fox family in the context of bone-associated cell metabolism and various bone diseases (Figure 1). In addition, we highlight the future research directions by identifying related novel biomarkers for cancer diagnosis and therapeutic targets.

## 2 Role of Fox factors in osteoporosis

**FIGURE 2:**
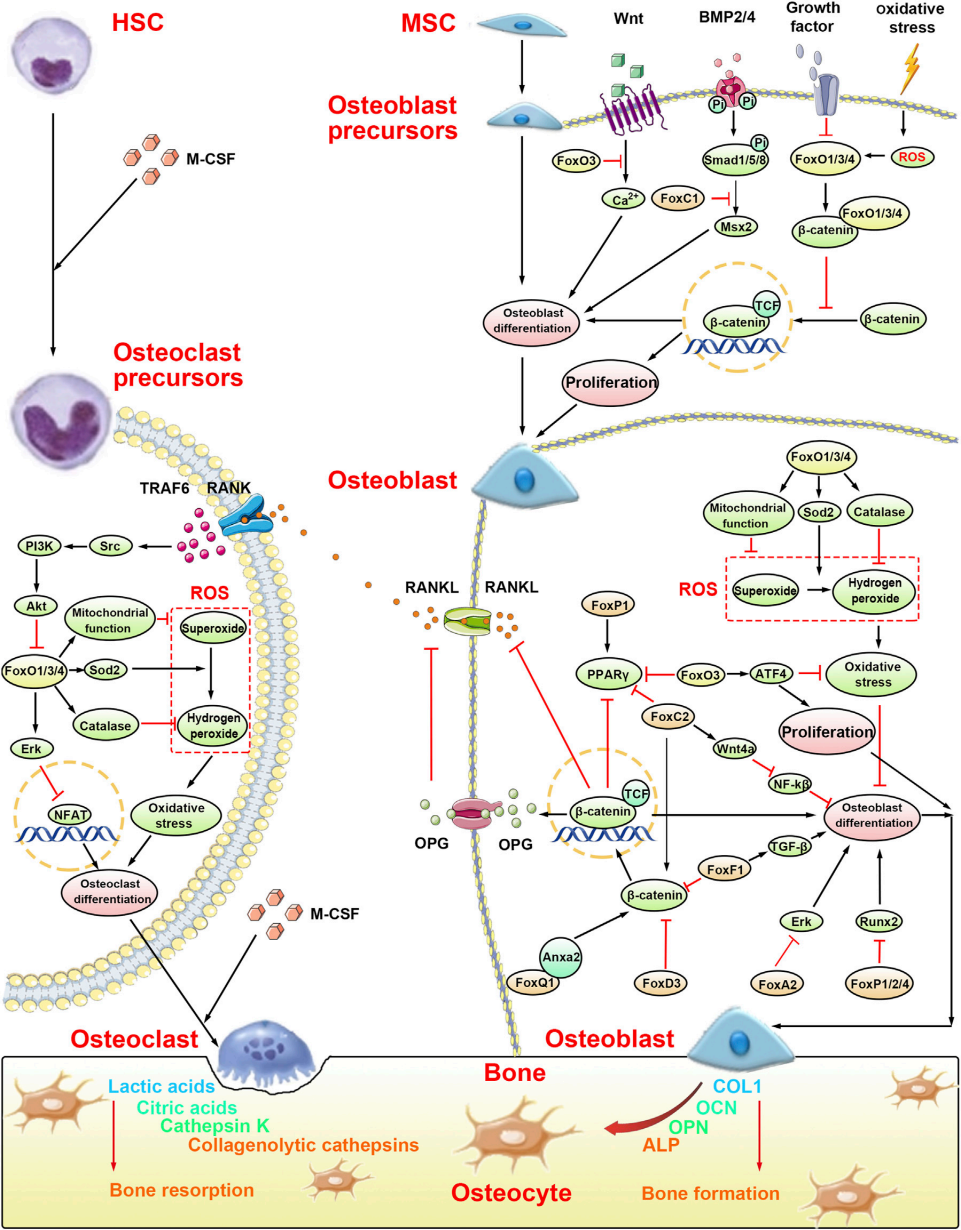
Molecular mechanisms of bone formation and bone resorption in osteoporosis by the Fox family. The occurence of osteoporosis is caused by the imbalance of bone formation, which is caused by osteoblasts, and bone resorption, which is caused by osteoclasts. Osteoblast precursors, originated from MSC, will be suppressed in osteoblast differentiation by FoxC1 and FoxO1/3/4. Meanwhile, FoxO1/3/4 can suppress the proliferation of osteoblast precursors by binding with β-catenin. In osteoblast, FoxA2, FoxC2, FoxD3, and FoxP1/2/4 suppress osteoblast differentiation. However, FoxO1/3/4 and FoxQ1 promote osteoblast differentiation. Interestingly, FoxF1 not only suppress osteoblast differentiation by the Wnt/β-catenin signaling pathway but also promote osteoblast differentiation by the TGF-β signaling pathway. Furthermore, FoxO3 can promote proliferation of osteoblast precursors by activating ATF4. Osteoclast precursors, originated from HSC, will be suppressed in osteoclast differentiation by FoxO1/3/4 through the Erk signaling pathway and ROS-dependent pathway.

Osteoporosis is a metabolic bone disease (Bellavia et al., 2021) caused by dyshomeostasis of bone metabolism. The dynamic balance of bone metabolism depends primarily on the interaction between osteoblasts, which synthesize the bone matrix, and osteoclasts, which absorb the bone matrix. This dynamic balance is essential for preventing bone disease in the human body (Huang et al., 2020). Recent studies have indicated a key role of the Fox family in the process of bone metabolism (Figure 2). Among these, most Fox factors have been shown to be involved in osteogenic differentiation via different signal pathways (Table 1 and Table 2); however, FoxO, FoxP, and FoxM1 play their respective roles in both osteoblast differentiation and osteoclast differentiation.

**TABLE 1:** The role of the Fox subfamilies in osteogenic differentiation.

Fox genes	Expression	Target genes/pathway	Effects on osteogenesis	Cells/animal model	Reference
FoxO1	Increased	Runx2, ALP, OCN	Promoted	C3H10T1/2 cells	Teixeira et al. (2010)
FoxO1	Increased	miR-424/FGF2, Runx2	Promoted	BMSCs	Li et al. (2017)
FoxO1/3/4	Deleted	PPARγ, Runx2, Osx, p66^shc^	Inhibited	Deletion of FoxO1/3/4 mice	Ambrogini et al. (2010)
FoxO1/3/4	Deleted	Wnt/β-catenin, cyclin D1	Promoted	Bipotential progenitors of osteoblast and adipocytes	Iyer et al. (2013)
FoxO3a	Increased	ALP, OCN, Runx2, LRP5, LRP6	Promoted	BMSCs	Sun et al. (2018)
FoxO3a	Increased	OCN, Runx2	Inhibited	MC3T3-E1 cells	Tang et al. (2019)
FoxC1	Decreased	Msx2, Runx2, ALP	Inhibited	C2C12 cells	Hopkins et al. (2016)
	Decreased	Runx2, osterix	Promoted	MC3T3 cells	Hopkins et al. (2016)
FoxC1	Increased	Msx2, Runx2, ALP	Promoted	C2C12 cells	Mirzayans et al. (2012)
FoxC1	KD	Msx2, ALP, OCN, Runx2	Promoted	O9-1 cells	Sun et al. (2013)
FoxC2	Increased	ALP, OCN, Cbfα1, Wnt/β-catenin, BSP, PPARγ2	Promoted	BMSCs	(Lin et al., 2016)
FoxP1	OE	Recombination signal-binding protein, ALP	Promoted	C3H10T1/2 cells	Li et al. (2017)
	KO	ALP, COL1A1, PPARG, CEBPA, FABP4	Inhibited	BMSCs	Li et al. (2017)
FoxP1/2/4	Decreased	Runx2	Promoted	Skeletal progenitor cells	Zhao et al. (2015)
FoxA2	KD	ERK, ALP, OPN, OCN, Runx2, Col1a1	Promoted	BMMSCs	Ye et al. (2018)
FoxD3	OE	OSX, TNAP, SOX9, OPN	Promoted	hESCs	Kamaldinov et al. (2018)
FoxD3	Increased	Wnt/β-catenin	Inhibited	BMMSCs	Huang and Chen, (2017)
FoxF1	KD	Wnt/β-catenin	Promoted	BMSCs	Shen et al. (2020)
FoxF1	Increased	TGF/β-catenin	Promoted	BMSCs	(Weng et al., 2019)
FoxM1	Decreased	RANKL/OPG, ALP	Promoted	PDLCs	Li et al. (2019)
FoxQ1	Increased	Wnt/β-catenin, ANXA2, ALP, OCN, OPG, Runx2	Promoted	BMSCs	Xiang et al. (2020)
FoxI1	Deleted	Fgf8	Inhibited	Foxi1 mutant embryo	Nissen et al. (2003)
FoxI3	Deleted	Pax8, Fgf3	Inhibited	Foxi3 mutant Mice	Edlund et al. (2014)
FoxL2	Deleted	GH/IGF1, SOX9	Inhibited	FoxL2^−/−^ mice	Marongiu et al. (2015)
FoxG1	Decreased	Osteocalcin, ALP	Inhibited	Osteoblasts	Uchida et al. (2018)

Note. OE, overexpression; KD, knock down; BMSCs, bone marrow mesenchymal stem cells; BMMSCs, bone marrow‐derived mesenchymal stem cells; PDLCs, periodontal ligament cells; hESCs, human embryonic stem cells; ALP, alkaline phosphatase; Runx2, Runt-related transcription factor 2; OCL, osteocalcin; PPARγ, proliferator-activated receptor γ2; msx2, msh homeobox 2; ERK, extracellular-regulated protein kinases; RANKL, receptor activator of nuclear factor-κB ligand.

**TABLE 2:** The role of the Fox subfamilies in osteoclast differentiation.

Fox genes	Expression	Target genes/pathway	Effects on osteoclastogenesis	Cells/animal model	Reference
FoxO1	Decreased	PI3K/AKT	Promoted	Mice with conditional loss	Bartell et al. (2014)
FoxO1	OE	Myc, ERK	Inhibited	RAW264.7 cells	Tan et al. (2015)
FoxO1	Decreased	NFATc1	Promoted	Bone marrow macrophages/RAW264.7 osteoclast precursors	Wang et al. (2015)
FoxM1	KD	RANKL/OPG	Promoted	RAW264.7 cells	Li et al. (2019)
FoxM1	Deletion	RANKL/OPG/RANK	Inhibited	Mice AtoMs	Hasegawa et al. (2019)
FoxP1	OE	INF-γ, MCP-1	Inhibited	FoxP1-overexpressing transgenic mice	Shi et al. (2008)
FoxP3	Increased	RANKL/OPG/RANK and IFNγ	Promoted	Osteoclast precursors	(Zaiss et al., 2007; Zaiss et al., 2010)

### 2.1 Fox factors with dual action on bone metabolism

#### 2.1.1 FoxO family

In mammals, FoxO family consists of four members: FoxO1, FoxO3, FoxO4, and FoxO6. Among these, FoxO1, FoxO3, and FoxO4 are expressed in the bone, while FoxO6 is confined to specific structures of the developing brain (Jacobs et al., 2003). In this review, due to the limitation of technology, some papers did not mention the specific member of the FoxO family but mentioned FoxO in general in their papers. Thus, in our paper, FoxO was represented as FoxO1/3/4. FoxO activity is mainly regulated by the PI3K/Akt pathway (Brunet et al., 1999) and posttranslational modifications (phosphorylation, acetylation, and ubiquitination) (Ambrogini et al., 2010). In addition, it can reduce the production of reactive oxygen species (ROS) by regulating antioxidant enzymes (superoxide dismutase and catalase) (Salih and Brunet, 2008) and upregulating mitochondrial function (Zorov et al., 2014) to mediate oxidative stress, thereby stimulating the differentiation of bone marrow mesenchymal stem cells (BMSCs) into osteoblasts and inhibiting their senescence (Sun et al., 2018) (Figure 2 “Osteoblast” part). Consistently, silencing of *FoxO1* was found to inhibit the expression of osteogenic markers, such as Runt-related transcription factor 2 (Runx2), alkaline phosphatase (ALP), and osteocalcin (OCN), even in the presence of strong osteogenic stimulants, resulting in decreased culture calcification (Teixeira et al., 2010). Moreover, mice with conditional deletions of *FoxO1*, *FoxO3*, and *FoxO4* showed profound loss of bone mass in calvaria, vertebrae, and femoral bone, which was caused by the upregulation of osteoblast apoptosis and oxidative stress (Ambrogini et al., 2010). In addition, three main FoxO isoform mice with deletion in growth plate chondrocytes showed a distinct elongation of the hypertrophic zone of the growth plate in the neonatal period; these mice showed severe skeletal deformities at older ages, indicating the importance of FoxO signaling in chondrocytes during endochondral ossification (Salih and Brunet, 2008).

However, studies have provided different insights on the function of FoxO family (Figure 2 “Osteoblast precursors” part). Increased oxidative stress was found to activate the FoxO family, which inhibited the Wnt/β-catenin signaling pathway through competing with T-cell factor transcription factor for binding with β‐catenin, and then decreased bone formation (Iyer et al., 2013). Moreover, overexpression of FoxO3 inhibited alterations in the expression of the calcium channel and decreased calcium absorption and calcium deposition, thereby preventing osteoblast differentiation (Tang et al., 2019). These studies suggest that the regulatory effect of FoxO on osteogenic differentiation is closely associated with the type of cell; for instance, in osteoblast precursors and osteoblasts, the FoxO family showed an absolutely opposite regulatory effect on osteoblast differentiation.

Similarly, contradictory findings have been reported about the regulatory effect of FoxO family on osteoclast differentiation (Figure 2 “Osteoclast precursors” part). In mice with conditional loss, receptor activator of nuclear factor-κB ligand (RANKL) was shown to induce activation of the Src pathway, subsequently activating the PI3K–Akt pathway, and downregulating FoxO1/3/4, thereby decreasing catalase, and increasing the accumulation of H_2_O_2_ and the levels of ROS (Bartell et al., 2014). This ultimately enhanced osteoclast formation, activation, and survival. Furthermore, Tan et al. found that the inhibitory effect of FoxO1 on osteoclast development was partly mediated by suppression of MYC and upregulation of extracellular-regulated protein kinases (Erk, Tan et al., 2015). However, in the study by Wang et al., FoxO1 deletion decreased osteoclastogenesis and RANKL-induced osteoclast activity in both experimental bone marrow macrophages and in RAW264.7 cells (Wang et al., 2015). These two contradictory results actually reflect the different effects of the FoxO family at different time periods on osteoclast differentiation; the long-term effect was associated with oxidative stress and aging, which enhanced osteoclast formation, and the short-term effect was associated with modulation of RANKL-stimulated osteoclast formation, which inhibited osteoclast formation.

Collectively, although there is no clear consensus about the ultimate effect of FoxO on osteoblast differentiation and osteoclast differentiation, we were able to identify the importance of FoxO transcription factors involved in the regulation of bone metabolism, including through antioxidative stress and the PI3K/Akt pathway, as potential therapeutic targets for osteoporosis. In particular, it seems that the PI3K/Akt pathway may be a coupling target of FoxO on osteoblast differentiation and osteoclast differentiation.

#### 2.1.2 FoxP family

The FoxP family consists of four members: FoxP1, FoxP2, FoxP3, and FoxP4 (Takahashi et al., 2009). Among these, FoxP1/2/4, which play a key role in the development of proper long bone in transgenic mice, are suggested to be negative regulators of Runx2 (Zhao et al., 2015) (Figure 2 “Osteoblast” part). Overexpression of FoxP1/2/4 in chondrocytes inhibits the endochondral ossification pathway and severely impairs chondrocyte hypertrophy and osteoblast differentiation (Zhao et al., 2015). In addition, FoxP1 significantly affected proliferator-activated receptor γ2 (PPARγ2) transcription, increasing lipogenic differentiation of mesenchymal progenitors at the cost of osteogenic differentiation (Wang et al., 2020) (Figure 2 “Osteoblast precursors” part). On the contrary, because of the dual role in regulating the fate switch and aging of MSCs, a study conducted by Li et al. found that *FoxP1* favors bone formation over adipogenesis and may be a potential target gene for the treatment of osteoporosis (Li et al., 2017). Meanwhile, loss of *Foxp2* in skeletal tissue also led to pleiotropic deficits in skull shaping and bone strengthening, indicating that *Foxp2* played a key role in the process of endochondral ossification (Xu et al., 2018). These different phenomena may be due to the difference in cells, tissues, and physiological microenvironment. FoxP1/2/4 play an important role in bone formation, in particular, FoxP1; however, further studies are required to unravel the complex regulatory effect on osteogenic, chondrogenic, and lipogenic differentiation.

Interestingly, FoxP family also plays a pivotal role in osteoclast differentiation. Osteoclasts originate from the monocyte lineage, and defects in monocyte differentiation are usually accompanied by disordered osteoclastogenesis (Dai et al., 2002). Upregulation of FoxP1 has been shown to result in impaired monocyte and macrophage function. Compared with wild-type mice, reduced total tartrate-resistant acid phosphatase (TRAP)-positive cells and decreased osteolytic viability were observed in transgenic mice overexpressing human FoxP1 under induction of macrophage colony-stimulating factor and RANKL (Shi et al., 2008). FoxP3, a transcription factor expressed by T-regulatory cells, is a spectrum master regulator of Treg cell development and suppressor activity (Deng et al., 2019). FoxP3 + Treg cells inhibit RANKL-induced osteoclastogenesis through various mechanisms that may be cytokine dependent, such as IL-4, IL-10, and TGF-β, or cell dependent via cytotoxic T-lymphocyte-associated antigen-4 contact (Zaiss et al., 2007). FoxP3 overexpression in mice resulted in decreased numbers of osteoclasts, resulting in reduced bone resorption activity; the reduced osteoclast numbers were not caused by an intrinsic defect in osteoclast differentiation. Nevertheless, FoxP3-deficient bone marrow increased local and systemic bone loss (Zaiss et al., 2010). Thus, the FoxP family inhibits osteoclast differentiation.

Collectively, the available evidence suggests a key role of the FoxP family in the prevention and treatment of osteoporosis through its involvement in bone metabolism and its protective effect against cellular senescence. However, there is a paucity of studies investigating the roles of the FoxP family in bone formation. Further studies are required to unravel the specific mechanisms and effects of FoxP on osteoblastogenesis and osteoclastogenesis.

#### 2.1.3 FoxM1

Inhibition of FoxM1 has been shown to enhance osteogenic differentiation of human periodontal ligament cells (PDLCs) (Li et al., 2019). Besides, the Wnt/β-catenin signaling pathway, an important pathway in osteogenic differentiation (Zhou et al., 2017; Huang et al., 2019), has been shown to be linked with FoxM1 in the field of oncology (Zhang et al., 2011; Gong and Huang, 2012; Chen et al., 2016). However, whether FoxM1 acts on the Wnt/β-catenin signaling pathway to regulate osteogenic differentiation has not been investigated.

There is no clear consensus on the regulatory role of FoxM1 in osteoclast differentiation. On the one hand, TRAP staining showed an increase in the number of multinucleated osteoblasts in Raw264.7 cells and upregulated RANKL/osteoprotegerin ratio in PDLCs when human PDLCs were inhibited with siRNA and specific inhibitor Siomycin A of FoxM1 cocultured with Raw264.7 cells (Li et al., 2019). On the other hand, FoxM1 deletion was found to partially inhibit synovial R3 cell osteoclastogenesis *in vitro*, and the bone degradation attenuated by tamoxifen-induced FoxM1 deletion was partly reversed by the overt transfer of FoxM1^+/+^ CX_3_CR1^+^ monocytes *in vivo* (Hasegawa et al., 2019). These findings suggest that deletion of FoxM1 inhibits the ability of osteoclast precursors to differentiate into osteoclasts both *in vivo* and *in vitro*.

There is obvious evidence of the involvement of FoxM1 in the regulation of bone metabolism and influencing the development of osteoporosis. However, the underlying mechanisms are not well understood due to a paucity of related studies.

### 1.2 Fox factors that affect osteogenic differentiation alone in bone metabolism

In addition to FoxO family, FoxP family, and FoxM1, several Fox subfamilies have been reported to be involved in the regulation of bone metabolism, which is limited to the regulation of osteogenic differentiation.

*FoxC* genes are pivotal in the regulation of bone development and cartilage formation (Chen et al., 2019; Yoshida et al., 2015; Xu et al., 2021). In particular, FoxC1 is an important regulator both in the initial steps of intramembranous osteogenesis (Hopkins et al., 2016) and in early and late endochondral ossification (Yoshida et al., 2015). Mice with spontaneous loss of function mutations (FoxC1^ch/ch^) die shortly after birth and exhibit skeletal abnormalities and defects (Hong et al., 1999). *In vivo*, FoxC1 exhibits different osteogenic differentiation effects on different cells (Hopkins et al., 2016). FoxC1 regulates osteogenic precursor cell differentiation and cranial bone development through its action on msh homeobox 2, a key regulator of bone formation and craniofacial skeletal development (Mirzayans et al., 2012) (Figure 2 “Osteoblast precursors” part); however, its ultimate effects are divergent (Rice et al., 2003; Mirzayans et al., 2012; Sun et al., 2013). Besides, FoxC2 acts on the Wnt signaling pathway to promote bone formation. On the one hand, FoxC2 directly activates the classical Wnt/β-catenin signaling pathway, increasing the expression of osteogenic markers, such as Runx2, COL1A1, OCN, and osteopontin, and inhibiting the expression of PPARγ2 (You et al., 2013; You et al., 2014; Zhou et al., 2019a). On the other hand, FoxC2 binds to the Wnt4 promoter and stimulates the nonclassical Wnt signaling pathway by activating the p38/MAPK pathway (Chang et al., 2007) and inhibiting NF-κB to promote differentiation and bone formation in BMSCs (Yu et al., 2014) (Figure 2 “Osteoblast” part).

*FoxI1/3* and *FoxL2* genes are also important regulators of bone development (Ohyama and Groves, 2004; Uda et al., 2004). Both FoxI1 deletion in zebrafish (Nissen et al., 2003) and FoxI3 mutants in mice (Edlund et al., 2014) cause severe structural defects of the facial skeleton, such as malformation and absence of the external ear and jaws (Edlund et al., 2015). FoxL2^−/−^ mice, which died in large numbers soon after birth (Uda et al., 2004), showed abnormal cranial, vertebral, and pelvic development, with bone loss and impaired cartilage formation (Marongiu et al., 2015).

In addition, knockdown of FoxA2 (FoxA2-KD) promotes osteogenic differentiation of BMSCs partially activating the ERK pathway (Ye et al., 2018) and overexpression of FoxQ1 promotes osteogenic differentiation of BMSCs through the Wnt/β-catenin pathway by binding with annexin a2^67^ (Figure 2 “Osteoblast” part). FoxG1 knockout (KO) osteoblasts exhibit lower mRNA expressions of Runx2, Osterix, and ALP; however, the underlying mechanism is still unknown (Kimira et al., 2017; Uchida et al., 2018).

There is inconsistency in the reported effects of FoxD3 and FoxF1 on osteoblast differentiation (Figure 2 “Osteoblast” part). Upregulation of FoxF1 during MOTS-c-induced osteogenesis activates the TGF-β pathway, thereby promoting fracture healing (Zeng et al., 2017). However, in another study, FoxF1 knockdown significantly increased osteogenic-specific gene expression and mineralization, which was associated with partial activation of the Wnt/β-catenin pathway (Shen et al., 2020). In ovariectomized (OVX) mice, knockdown of FoxF1 with siRNA significantly reduced OVX-induced bone loss by enhancing bone formation, suggesting that FoxF1 may be a marker factor for bone formation and a therapeutic target for postmenopausal osteoporosis (Shen et al., 2020). The mRNA and protein expressions of FoxD3 were upregulated in bone marrow‐derived mesenchymal stem cells treated with IL-1β. Huang et al. pointed out that FoxD3 may mediate transcriptional activation of miR-496 triggered by IL-1β, thereby repressing the Wnt/β-catenin signaling pathway and reducing osteoblast differentiation (Huang and Chen, 2017). Interestingly, a study by Kamaldinov et al. showed that overexpressed FoxD3 may enhance osteogenesis in human embryonic stem cells via the endochondral ossification pathway (Kamaldinov et al., 2018).

The above evidence demonstrates that Fox factor plays a key role in osteoporosis pathology by acting on both osteoblast differentiation and osteoclast differentiation or by influencing osteoblast differentiation alone to regulate bone metabolism.

## 3 Role of Fox factors in osteoarthritis

Osteoarthritis is a chronic joint disease characterized by degenerative changes in joint cartilage. Aging and inflammation are the main risk factors for osteoarthritis (Duan et al., 2020). Studies have indicated the involvement of the FoxO family and FoxM1 in the development of osteoarthritis. In an *in vitro* experiment, upregulation of FoxO increased the expressions of autophagic genes (*Map1lc3b*, *Atg4b*, *Becn1*, *Gabarapl1*, *and Bnip3*), which could prevent aging, protecting cartilage by mediating apoptosis, and elimination of ROS (Charlier et al., 2016). Moreover, by upregulating mitochondrial function and reducing intracellular ROS (Bartell et al., 2014), and reducing the production of inflammatory factors (chemerin) and cartilage-degrading enzymes (Akasaki et al., 2014), FoxO1 delayed chondrocyte senescence and reduced chondrocyte apoptosis, respectively. Matsuzaki et al. found that FoxO1 acts synergistically with TGF-β to activate recombinant proteoglycan 4 expression, which is essential for maintaining the integrity of the superficial cartilage region. FoxO triple KO mice (AcanCreERT-TKO) exhibited complete cartilage defects, more severe synovial inflammation, and subchondral bone changes after administration of tamoxifen for 5 months (Matsuzaki et al., 2018). FoxM1 impairs chondrocyte viability and accelerates the development of osteoarthritis. In a lipopolysaccharide-induced osteoarthritis model, FoxM1 was shown to bind with signal transducer and activator of transcription3 (STAT3) in the nucleus, leading to its upregulation and phosphorylation, impairing chondrocyte viability. Knockdown/silencing of FoxM1 inhibited the production of inflammatory factors and NF-κB activation, enhancing cell viability in an osteoarthritis model (Zeng et al., 2019; Zeng et al., 2021). Moreover, miR-877-5p was shown to improve chondrocyte function by inhibiting FoxM1 in both *in vivo* and *in vitro* experiments (Zhu et al., 2020a).

## 4 Role of Fox factors in rheumatoid arthritis

Rheumatoid arthritis is a chronic joint disease characterized by persistent synovitis and associated damage to the articular cartilage and subchondral bone. Smoking is a major environmental risk factor for rheumatoid arthritis (Scott et al., 2010). Activation of the PI3K–Akt axis by differentially expressed miRNAs in smokers leads to rheumatoid arthritis, in part through FoxO inactivation (Reedquist et al., 2006; Wasén et al., 2020). However, rapid downregulation of FoxO1 in rheumatoid arthritis fibroblast-like synoviocytes in response to IL-1β or PDGF stimulation is independent of Akt and results from accelerated c-Jun N-terminal kinase (JNK)-mediated degradation of FoxO1 mRNA (Grabiec et al., 2015). Moreover, autophagy protects chondrocytes from glucocorticoid-induced apoptosis through upregulation of the ROS/Akt/FoxO3 signaling pathway (Shen et al., 2015). In addition to the FoxO family, FoxM1, which is related to damage to subchondral bone, may also play a role in the pathogenesis of rheumatoid arthritis (Hasegawa et al., 2019). High energy is required for osteoclastogenesis under arthritic conditions, and FoxM1 directly drives mitochondrial biogenesis (De Luca et al., 2015), promoting differentiation of AtoMs into osteoclasts. *In vivo*, FoxM1 inhibition alleviated not only articular bone destruction but also joint inflammation (Hasegawa et al., 2019). Thus, thiostrepton, a direct inhibitor of FoxM1, which inhibits FoxM1 binding to genomic target sites (Hegde et al., 2011), may be a new approach to rheumatoid arthritis treatment.

## 5 Role of Fox factors in intervertebral disc homeostasis and intervertebral disc degeneration

Intervertebral disc (IVD) is a fibrocartilaginous tissue that lies between two vertebrae and functions as a shock absorber. It includes the jelly-like nucleus pulposus, the surrounding fibrocartilaginous annulus fibrosus, and the cartilaginous endplate anchoring the IVD to the corpus vertebrae (Kamali et al., 2021). Intervertebral disc degeneration (IDD), the major cause of chronic low back pain (Maher et al., 2017), was recently shown to be closely related with the Fox family. FoxO is required for intervertebral disc homeostasis during aging, and its deficiency promotes disc degeneration (Alvarez-Garcia et al., 2018). In a study by Xia et al., upregulation of FoxO3 promoted proliferation and inhibited apoptosis of nucleus pulposus cells in IDD (Xia et al., 2021). Moreover, FoxO3 was shown to retard IDD by antioxidative stress (Zhou et al., 2019b). Furthermore, FoxA1^−/−^, FoxA2^c/c^, and ShhcreER^T2^ double mutant animals showed severely deformed nucleus pulposus, increase in cell death in the tail, decreased hedgehog signaling, defects in the notochord sheath, and aberrant dorsal–ventral patterning of the neural tube (Maier et al., 2013). Recently, Zhou et al. revealed that FoxA2 regulates the type II collagen-induced nucleus pulposus-like differentiation of adipose-derived stem cells via activation of the Shh signaling pathway (Zhou et al., 2018).

## 6 Role of fox factors in bone tumors

### 6.1 Osteosarcoma

Osteosarcoma (OS) is the most common primary malignant tumor of bone, and it occurs mainly in children and adolescents (Kansara et al., 2014). Recently, researchers have conducted in-depth studies on the mechanisms of FoxO, FoxM1, and FoxC2 in the development of OS and the drugs that target these Fox factors. First, FoxO induces G1 cell cycle arrest, apoptosis, and DNA repair (Nakamura et al., 2000), and is considered to be a tumor suppressor (Niedan et al., 2014). FoxO1 expression was absent or low in OS cells, and upregulation of FoxO1 expression induced OS cell cycle arrest and apoptosis, and reduced the number of colonies (Guan et al., 2015). For instance, E2F transcription factor 1 induces the expression of FoxO1 and interacts with it to activate the target gene apoptotic protease-activating factor-1, promoting apoptosis in U2OS cells (Shats et al., 2013). Therefore, a number of drugs targeting FoxO are already being used in studies for OS treatment (Herman et al., 2020). Grifolin inhibits the PI3K/Akt/FoxO1 pathway in human OS cells suppressing their proliferation and inducing apoptosis (Jin et al., 2007). Brazilin was shown to increase the expression of autophagy-related genes and promote death of human OS cells (MG-63 cells) by interfering with the steady-state phosphorylation of the Ser7 site of FoxO3a by Ca^2+^ (Kang et al., 2018).

Second, FoxM1 is associated with tumor cell proliferation, migration, invasion, and angiogenesis, suggesting that FoxM1 is an oncogenic factor (Halasi and Gartel, 2013). FoxM1 is highly expressed in human OS tissues and cell lines, and downregulation of FoxM1 expression was found to inhibit the viability, migration, and invasive growth of OS cells (Zhu et al., 2020b). Recent studies have shown that avasimibe (Wang et al., 2019a), diallyl disulfide (Li et al., 2018), thiostrepton (Cai et al., 2020), and some miRNAs [including miR-134 (Li et al., 2018), miR-370 (Duan et al., 2015), miR-216b (Wang et al., 2019b), and miR-197 (Sun et al., 2020)] inhibit the proliferation and invasive growth of OS cells by directly or indirectly downregulating FoxM1 expression.

Third, FoxC2, a transcription factor involved in epithelial–mesenchymal transition (EMT), is defined as a carcinogenic factor (Kalluri, 2009). Silencing of FoxC2 expression attenuated anchored nondependent growth of OS cells and reduced the invasive ability, which may be related to downregulation of C-X-C motif chemokine receptor 4 (Gozo et al., 2016). FoxC2 can regulate chemoresistance in OS; for instance, siRNA transfection-mediated knockdown of FoxC2 increased the sensitivity of two OS cell lines to doxorubicin (Zhang et al., 2017).

Lastl, the FoxP family was found to be closely related with OS (Gascoyne et al., 2015; Yin et al., 2017; Li et al., 2021). In the study by Li et al., FoxP1 was shown to promote proliferation, tumor sphere formation, migration and invasion, and inhibit anoikis by FOXP1 overexpression and knockdown in OS cell lines (Li et al., 2021). In 143B OS cells with minimal endogenous expression, FOXP2 induced by growth arrest is required for upregulation of p21^WAF1/CIP1 107^. Upregulation of miR-491-5p suppressed proliferation of human OS cells and induced apoptosis by targeting FoxP4 (Yin et al., 2017).

### 6.2 Ewing sarcoma

Ewing sarcoma (ES) is a rare and highly aggressive cancer that occurs primarily in the bones and surrounding tissues of children and adolescents. It is the second most common primary malignant bone tumor in children and adolescents (Balamuth and Womer, 2010; Dowless et al., 2018). The pathogenetic mechanism of ES is still not well understood. Studies have focused on the downstream target genes of the oncogenic transcription factor EWS/FLI1 (Balamuth and Womer, 2010). FoxO1, FoxM1, and FoxQ1, members of the Fox transcription factor family were shown to be three potent targets downstream of EWS/FLI1 (Cidre-Aranaz and Alonso, 2015).

FoxO1, as previously mentioned, is a tumor-suppressor factor with low expression in ES caused by the repressive effect of EWS/FLI1 binding to the FoxO1 promoter (Yang et al., 2010). Besides, negative regulation of FoxO1 activity and nuclear localization, caused by CDK2 (a negative regulator of EWS–FLI1-induced FoxO1 transcriptional activity) and PI3K/Akt-mediated FoxO1 phosphorylation, accelerated proliferation and promoted soft agar colony formation in two Ewing sarcoma cell lines (A673sh and SK-N-MC) (Niedan et al., 2014). Thus, methylseleninic acid, a drug reported to induce elevated expression of FoxO1 in ES cells, apoptosis of ES cells, and significant reduction of tumor growth in an orthotopic mouse xenotransplantation model, may be a potential target drug for Ewing sarcoma (Cidre-Aranaz and Alonso, 2015).

FoxM1, an oncogenic factor, is highly expressed in Ewing sarcoma and cell lines. Reduction of FoxM1 expression impairs the ability of Ewing cell lines to grow in an anchorage-independent manner (Christensen et al., 2013). Based on this, some studies have shown that thiazole antibiotics and proteasome inhibitors represented by thiostrepton (Gartel, 2011) and Siomycin A (Bhat et al., 2009) may have a role in the treatment of Ewing sarcoma by inhibiting FoxM1 expression.

High expression of FoxQ1 was detected in human ES lines KH and EWS. FoxQ1 may be associated with enhanced activation of downstream target genes through interaction with EWS–FLI1 at the N-terminal end of EWS, thereby promoting ES proliferation (Shimizu et al., 2018).

### 6.3 Metastatic bone tumors

During EMT, elevated FoxI3 expression correlates with the dedifferentiated state and motility of cells (Ye et al., 2015). Recent studies have demonstrated high expression of FoxI3 in bone metastases from prostate and breast cancers, suggesting that FoxI3 may promote bone metastasis and tumor growth and infiltration in the bone (Haider et al., 2016; Mukherjee et al., 2018). In addition, knockdown of FoxA2 inhibited bone metastasis of prostate cancer. Osteolytic lesions and tumor incidence were significantly lower in the tibia of FoxA2-suppressed mice compared with the control group (Connelly et al., 2020), which may be related to decreased expression of parathyroid hormone-related protein, a major factor mediating cancer-induced osteoclast production (Martin and Johnson, 2019), encoded by the *PTHLH* gene (Connelly et al., 2020).

## 7 Role of Fox factors in hereditary bone diseases

FoxC1 mutation is associated with Axenfeld–Rieger syndrome, an autosomal dominant disorder characterized by major skeletal abnormalities, such as mild craniofacial deformities, including forehead protrusion, limb hypertrophy, anterior sphenoid, and maxillary hypoplasia (Seifi and Walter, 2018; Chen et al., 2019). Heterozygous mutation in *FoxL2* is associated with blepharophimosis-ptosis-epicanthus inversus syndrome, an autosomal dominant disorder that manifests primarily as eyelid and mild craniofacial defects (Jin et al., 2007).

## 8 Conclusion and perspectives

The role of Fox factors in skeletal development and skeletal dynamic homeostasis has attracted much academic attention. The role of Fox factors as effectors in many signaling pathways and the associated regulatory mechanisms, including their actions on downstream targets, is an emerging research hotspot. Uncontrolled expression of Fox factor can lead to a variety of bone diseases, such as osteoporosis, osteoarthritis, rheumatoid arthritis, and bone tumors. Regulation of Fox expression has important clinical implications for the prevention and treatment of these bone diseases. In the context of progressive population aging and increase in the number of elderly patients with bone diseases (such as fractures, osteoporosis, and rheumatoid arthritis), development of new and more effective treatments is a key imperative. The role of the Fox gene family represents an entirely new area of research in bone metabolism that is expected to address this challenge.

Although Fox factors have been identified to play a pivotal role in the maintenance of bone homeostasis, much of the contemporary research is limited to cellular and animal studies. The use of drugs targeting Fox factors in the treatment of bone tumors has been attempted; however, there are few reports of clinical efficacy. Apart from this, development of targeted drugs for Fox factors in the clinical treatment of other skeletal diseases is still at a theoretical stage. Therefore, clarifying the prospects for clinical application of the Fox factors and development of Fox-targeted drugs represent key areas of future research.

## Glossary

Fox: forkhead box
SIRT: sirtuin
WNT: wingless and int-1
BMP: bone morphogenetic protein
Smad: drosophila mothers against decapentaplegic
PI3K: phosphatidylinositol 4,5-bisphosphate 3-kinase
TGF-β: transforming growth factor-β
MAPK: mitogen-activated protein kinase
NF-κB: nuclear factor-kappa B
ROS: reactive oxygen species
BMSCs: bone marrow mesenchymal stem cells
Runx2: Runt-related transcription factor 2
TCF: T-cell factor
RANKL: receptor activator of nuclear factor-κB ligand
Erk: extracellular-regulated protein kinases
PPARγ2: proliferator-activated receptor γ2
TRAP: tartrate-resistant acid phosphatase
M-CSF: macrophage colony-stimulating factor
Tregs: T-regulatory cells
IL: interleukin
CTLA-4: cytotoxic T lymphocyte-associated antigen-4
OPG: osteoprotegerin
PDLCs: periodontal ligament cells
Msx2: msh homeobox two
COL1A1: collagen type I a 1
OCN: osteocalcin
OPN: osteopontin
ANXA2: annexin a2
Osx: osterix
ALP: alkaline phosphatase
OVX: ovariectomized
hESCs: human embryonic stem cells
Prg4: recombinant proteoglycan four
OE: overexpression
KD: knockdown
KO: knock out
LPS: lipopolysaccharide
STAT3: signal transducer and activator of transcription3
FLS: fibroblast-like synoviocytes
JNK: c-Jun N-terminal kinase
IDD: intervertebral disc degeneration
OS: osteosarcoma
E2F1: E2F transcription factor 1
APAF1: apoptotic protease activating factor-1
CXCR4: C-X-C motif chemokine receptor four
ES: Ewing sarcoma
MSA: methylseleninic acid
PTHLH: parathyroid hormone-related protein
ARS: Axenfeld–Rieger syndrome
BPES: blepharophimosis-ptosis-epicanthus inversus syndrome
